# Managing Lymphedema Induced by Lymphatic Filariasis: Implementing and Improving Care at the Individual and Programmatic Levels

**DOI:** 10.4269/ajtmh.23-0905

**Published:** 2024-07-30

**Authors:** Charles D. Mackenzie, D Ramaiah Kapa, Suma Krishnasastry, Jan Douglass, Achim Hoerauf, Eric A. Ottesen

**Affiliations:** ^1^ Coalition for Operational Research on Neglected Tropical Diseases (COR-NTD), Task Force for Global Health, Atlanta, Georgia;; ^2^ The END Fund, New York, New York;; ^3^ Consultant Lymphatic Filariasis Epidemiologist, Pondicherry, India;; ^4^ Filariasis Research Unit, WHO Collaborating Center for LF MMDP, Government T. D. Medical College Hospital, Kerala University of Health Sciences, Alappuzha, India;; ^5^ Division of Tropical Health and Medicine, James Cook University, Townsville, Australia;; ^6^ Institute for Medical Microbiology, Immunology and Parasitology, University Hospital of Bonn, Bonn, Germany;; ^7^ German Center for Infection Research (DZIF), Partner Site Bonn-Cologne, Germany

## Abstract

Providing and improving the care of patients suffering from lymphedema remains an essential goal for the clinical management of populations affected by lymphatic filariasis. Although the Essential Package of Care (EPC) recommended by the WHO leads to important positive benefits for many of these lymphedema patients, it is important to continue to address the challenges that remain both in quantifying these effects and in ensuring optimal care. This report, based on the authors’ scientific and field experience, focuses on the impact and significance of lymphedema, its clinical presentation, current treatment approaches, and the importance of lymphedema care to the Global Program to Eliminate Lymphatic Filariasis. It emphasizes specific practical issues related to managing lymphedema, such as the importance of beginning treatment in the condition’s early stages and the development of effective approaches to assess patients’ progress toward improving both their clinical status and their overall quality of life. Priorities for research are also examined, particularly the need for tools to identify patients and to assess disease burden in endemic communities, the creation of EPC accessibility to as many patients as possible (*i.e.*, targeting 100% “geographic coverage” of care), and the empowerment of patients to ensure the sustainability, and ultimately the provision of care from sectors of the national public health systems of endemic countries.

## INTRODUCTION

Infection with parasitic worms of the “lymphatic filariasis (LF) group” (*Wuchereria bancroft*i and the *Brugia* species) can result not only in the very recognizable clinical pathologies of lymphedema and hydrocele but also in more subtle physical changes such as abnormalities of the deep lymphatic system and renal function. Of the more prominent clinical manifestations, hydroceles are most directly addressable because with good surgical practice this condition is generally correctable. The other predominant manifestation is lymphedema, which presents a much greater challenge in terms of providing care that can reverse, or at least slow, the progression of the disease. It is this markedly debilitating, disfiguring presentation that leads to the most recognizable hallmarks of LF infection (e.g., grossly swollen limbs, multiple skinfolds, extensive dermal changes, and ultimately “elephantiasis”^†^), all associated with severe stigma and psychosocial consequences in affected individuals.^1^^,^^2^

**Figure 1.**
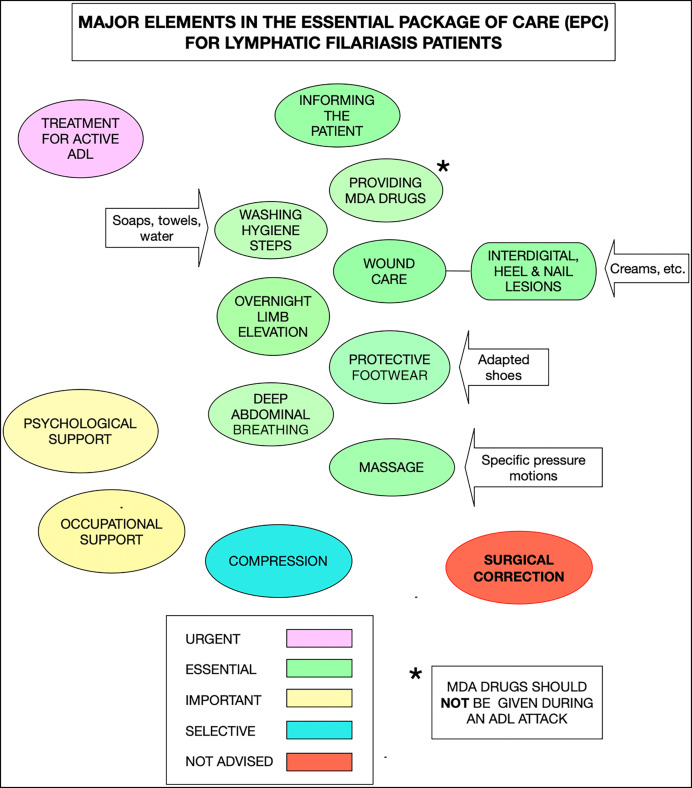
The elements of the Essential Package of Care (EPC) for LF lymphedema patients. ADL = adenolymphangitis; LF = lymphatic filariasis; MDA = mass drug administration.

The principal challenges to improving the management of patients’ lymphedema include not only the need to better understand the pathogenesis of filarial lymphedema, which might offer new treatment approaches, but also to tailor currently available treatments for use in the often-difficult field conditions in which many such patients live. It is important to recognize that the lymphedema resulting from this parasitic infection has distinct physical pathologies affecting vessels, tissues, and organs and that each of these components is a possible target for new therapies and approaches to improve the WHO’s recommended Essential Package of Care (EPC) (Figure 1) for lymphedema treatment.^3^^,^^4^ Despite the extensive and laudable successes many countries have had with the mass drug administration (MDA) elements of the Global Program to Eliminate Lymphatic Filariasis (GPELF) that have interrupted (and possibly eliminated) the transmission of filarial infections, many countries still need to address GPELF’s second target: alleviating the suffering associated with LF through targeted programs of morbidity management and disability prevention (MMDP).^5^ Improving the tools and strategies to address these MMDP issues is critical for assisting LF-endemic countries in reaching their MMDP goals. These targets remain essential to fulfilling their national commitment to ensure the ultimate success of GPELF.^6^^,^^7^

## LYMPHATIC FILARIASIS CLINICAL DISEASE AND ITS IMPORTANCE

It is the lymphatic disease manifestation itself, with striking images of the most severe and unfortunate clinical consequences (chronic lymphedema, elephantiasis), that has been the principal driver in motivating society to create a major global initiative to eliminate LF infection as a public health problem.

### Community awareness of lymphatic filarial disease.

Before GPELF began, there was often widespread misunderstanding of the fundamental causes of the clinical changes among many affected individuals and their endemic communities; indeed, early clinical stages of hydrocele and lymphedema were often ignored or misinterpreted because the fear of stigmatization led to keeping the presence of these conditions secret.

Learning that LF is a mosquito-borne worm infection and that appropriate care *can* be made available has been a major stimulus for patients’ willingness both to be identified as having the disease and then to seek care. Importantly, such understanding is most effective if it is shared community-wide and not just confined to patients and healthcare workers. Availability and active provision of care to affected community members encourages whole communities to participate in the MDA efforts of the GPELF,^8^ which are principally focused on interrupting parasite transmission (i.e., preventing future clinical disease) and not on the immediate amelioration of current LF disease. Knowing that care is available for those in need gives endemic populations confidence that the national LF elimination program is concerned about affected individuals and provides useful and valuable services for their communities through both MDA and MMDP. In Tanzania, for example, during the first year of MDA in 2001 many people were unwilling to take the offered medicines as they could not see that people with LF disease in the community were also being cared for; however, after care was visibly provided to affected community members during the following year, the subsequent MDA resulted in a much higher level of population treatment coverage.^9^

### Numbers of patients with filarial lymphedema.

Another important explanation for the inappropriately low number of reported lymphedema cases is likely the actual method itself for assessing their presence: namely, when being carried out either independently, or as an adjunct to MDA activities, a considerable number of cases may be missed. Indeed, recent studies comparing standard clinical reporting strategies with reporting by mobile phone–based surveillance systems operated by community health volunteers in Tanzania,^10^ Malawi,^11^ Ethiopia,^12^ and Ghana^13^ have shown that the latter approach was able to generate a more accurate assessment of lymphedema cases in both rural and urban areas. For example, the Interactive Voice Response System used in Ghana and arranged through a local mobile phone company, allowed structured reporting by community health volunteers without the need for even writing text messages; its use has led to the accurate reporting of far more lymphedema cases than did acquiring figures during MDA activities.^13^

The optimal, practical method for ascertaining the number of lymphedema cases is the creation of a house-to-house morbidity census (HHMC). Although the cost of this approach is high in some situations, the method is likely to have the greatest accuracy.^10^ In addition to directly identifying those infected, these household surveys can provide leads to other individuals in the community who might be affected but who are reticent about sharing their personal information directly with the surveyor. It is important to inform the community that the reason for identifying these patients is to be able to provide care that is now available for all those affected. Indeed, once a patient has been identified, it is incumbent upon a country’s national LF activities to provide the promised care as soon as possible; otherwise, not only will the patients’ care be compromised, but their community’s belief/faith in GPELF itself may also be lost.

An issue that further complicates the estimation of numbers of filarial lymphedema cases occurs in those regions where podoconiosis and LF coexist, principally in Africa but also in a small number of provinces in India, Indonesia, and elsewhere.^14^ Because the prevalence of podoconiosis in most cases is either unknown or represents a small fraction of that for LF and because the basic package of care for both causes of lymphedema is very similar, in countries where this etiologic confusion exists the practical approach to patient management has generally been to combine the care programs for both conditions as much as possible.^15^

Given the difficulties in identifying the total number of patients with filarial lymphedema,^16^ only approximations of the true number of individuals in need of care have been possible to date. Comprehensive assessment of the global literature in 1996,^17^ just prior to development of GPELF, led to the approximation that of the total number of individuals with LF infection, one-third could be expected to have clinical manifestations, with ∼30% of that one-third manifesting as lymphedema/elephantiasis and the remainder as hydroceles in men. When GPELF began, the number of patients with filarial lymphedema was crudely estimated at 18 million, and those with hydrocele at 30 million.^16^ As GPELF made steady progress, various studies estimated the impact of MDA intervention on chronic disease burden (the number of clinical cases). In 2014, 14 years after GPELF began, it was believed that 96 million LF infections had been cured, including 18.7 million hydrocele cases and at least 5.5 million lymphedema sufferers; it was suggested that 19.4 million hydrocele cases and almost 17 million cases of lymphedema remained.^17^ Modeling estimates in 2022 reported that GPELF (i.e., from 2000 to 2020) had prevented 44.3 million cases of chronic disease, including 16.9 million lymphedema cases and 27.3 million hydrocele cases.^18^ It was also suggested that in 2020, 16 million cases of hydrocele and 15 million cases of lymphedema remained. These authors also underscored that their models indicated that by 2020, without GPELF, there would likely have been at least 69 million cases of chronic disease globally, comprising 25 million lymphedema and 44 million hydrocele cases.

To restate, the actual number of clinical LF cases is hard to obtain and will not truly be known until all endemic countries complete their field inquiries and burden assessments; this figure is much needed as most of the existing lymphedema cases will require EPC to alleviate their suffering. Importantly, to best describe the success of GPELF in mitigating LF disease, especially for general advocacy and for collection and presentation of data, it is important to address 1) how many cases of infection and disease have been prevented, 2) how the disease’s age distribution has changed, and 3) how the reduction in infection levels in the population has led to decreases in new LF clinical cases and disease prevalence across different regions and age groups.

#### Economic burden.

The economic burden that the presence of lymphedema inflicts on patient, family, village, and country^19^ is an important metric for advocacy and for developing global initiatives such as those related to the United Nations’ Sustainable Development Goals.^20^^,^^21^ Previous estimates of the number of disability-adjusted life years (DALYs) associated with filarial lymphedema have varied, depending primarily on the origin of the data used for each analysis. Prior to GPELF, it was estimated that LF was responsible for 5.25 million DALYs, of which 1.75 million were attributed to lymphedema. The total economic burden due to acute episodes and chronic disease was estimated at USD 5.8 billion annually, of which USD 1.7 billion was due to lymphedema alone.^22^ Previously, issues such as mental health were not recognized as being important to include, but that all changed when Ton et al.^23^ emphasized the importance of depressive illness as a prevalent disability among patients with chronic LF. They estimated that, based on disease burden figures available in 2012, 5.09 million DALYs were associated with LF mental health burden alone and another 0.23 million DALYs were attributable to caregivers—in total, almost two times higher than the estimate of 2.78 million DALYs associated with LF disability highlighted in the Global Burden of Disease 2010 study.^24^ It has been projected that because of the 20 years of GPELF (2000–2020), 244 million DALYs were averted, 38% of them attributed to the prevented lymphedema.^25^^,^^26^ In addition, because many countries have now been validated as having successfully eliminated LF as a public health problem, it is expected that the overall economic burden has been further reduced because no new cases of LF infection will arise in these areas.

There are obviously a wide range of patients’ daily activities that are impacted by having lymphedema; these include loss of work with consequent loss of income, inability to support their families adequately, and basic personal challenges such as mobility, inability to ride bicycles, and similar activities. Although studies have been carried out on some of these aspects,^22^^,^^27^ further studies that focus on specific situations facing LF lymphedema patients in different environments (i.e., urban, rural, riverine, or mountainous areas) are likely to be informative.

### Consequences to patients, their families, and other caregivers.

The health consequences of LF for affected individuals increase as their lymphedema worsens. The episodic swelling and transient multi-day attacks of acute adenolymphangitis (ADL; also known as “acute attacks”) are tolerated in the early stages, but as they increase in frequency, intensity, and duration with progression of the disease, they become very much more problematic for the patient. Indeed, these acute attacks are commonly the prime reason patients actively seek medical help. With more severe lymphedema, it is not uncommon for patients to concern themselves primarily with preventing and treating ADLs specifically—an indication that although they might have become accustomed to having a disfigured limb, the acute attacks are still the most intolerable part of their LF disease.^2^^,^^9^ Patients who are most severely affected can even have multiple ADLs in a month, with devastating effects on their lives and on the lives of their families, including a negative impact on their socioeconomic status through decreased ability to work.^27^^,^^28^ A common statement by patients is that the lymphedematous limb feels abnormally heavy; after implementation of the care package, however, patients perceive relief that often leads to their expressing, with great satisfaction, that the affected limbs just “do not weigh as much anymore,” even without any externally apparent reduction in limb size.

For lymphedema patients, quality of life (QOL) has been assessed using different protocols, including some specifically modified for LF patients and others modified to include dermatological conditions (e.g., Dermatological Quality of Life Index- DQLI), one of the latter protocols having been carried out in Ethiopia where patients with podoconiosis, LF lymphedema, or leprosy were compared.^29^^–^^31^ Arguably, these QOL/DLQI protocols do not adequately cover the full range of daily activities in the lives of filarial lymphedema patients, as they only use either standard QOL questionnaires or the slightly less general, dermatologically oriented DLQI that was used in Ghana.^32^^,^^33^ There are also significant variations among endemic communities, including poor mental health concerns and personal stresses that are commonly found to be major factors impacting patients’ QOL.^23^^,^^33^^,^^34^ For Shilpa et al.,^35^ however, it was the lack of physical mobility, rather than poor mental health, that was the major factor in reducing the QOL of lymphedema patients in southern India. This finding is perhaps reflective of the comparatively strong patient support system available in southern India. There is no doubt, however, that the QOL for those with filarial lymphedema becomes increasingly worse as their condition worsens, with mental health, mobility, social interactivity, and wage earnings all being markedly compromised.^29^^,^^34^

Family members and other caregivers are also significantly impacted by the presence of clinical filariasis in the family, whether by directly assisting in hygiene and limb care activities, by helping in the daily tasks that the patient is unable to perform, or by sharing the psychological distress.^36^ When assessing the QOL of individuals suffering from lymphedema, the effects of the disease on family and caregivers must also be considered.

## UNDERSTANDING THE DEVELOPMENT OF FILARIAL LYMPHEDEMA

The underlying cellular, tissue, and organ changes associated with filarial lymphedema are still not well understood, and much of the knowledge we have of the pathogenesis in humans is inferred from experimental animal models, from patients evaluated with noninvasive imaging techniques (lymphoscintigraphy and ultrasonography), from hematology/serology testing, or from the relatively infrequently available biopsy material from surgical interventions. The direct presence of living parasites alters local tissue responses, especially in the early stages of infection, as emphasized by the informative studies of Shenoy et al.^37^ and Douglass et al.,^38^ who demonstrated clearly that the early-stage lymphatic pathology found in children with active filarial infections can be reversed by MDA drugs that remove the offending parasites. Similarly, experimental models, such as those of *Brugia* infections in cats^39^ and dogs,^40^ have shown many similarities with pathogenic findings in humans and have emphasized the interacting roles of the parasite, inflammation, and other cellular responses in early disease. Indeed, it is highly likely that the condition now recognized as filariasis-induced lymphedema involves a multifaceted complex of factors that affect both its pathogenesis and its treatment. Unfortunately (or perhaps fortunately!), it can be argued that the full range of mechanisms and components involved may never be fully elucidated because of the current, very successful efforts to reduce LF infection intensity and distribution worldwide.^41^ However, it still needs to be remembered that many of the lymphedema cases that already exist will continue to require care and thus will serve as a catalyst for finding new, improved treatments.

### Clinical presentation.

The clinical presentation of filarial lymphedema has been well described, along with several different staging systems for judging a patient’s status.^42^^,^^43^ From both clinical and patient perspectives, it is recognized as swelling of a limb with or without the occurrence of intermittent pain and enlargement of the draining lymph nodes (especially during an acute attack). In the early stages of lymphedema, the swelling often disappears overnight, especially if the limb is elevated during sleep.

As time passes, the swelling of the limb remains throughout the day and night, and the persistent accumulation of fluid in the subcutaneous and deeper tissues induces a decrease in the ability of the skin to maintain homeostasis. As a result, visibly worsening dermal changes occur, including hyperkeratosis, subdermal fibrosis, deep skinfolds, and deterioration of the skin initially in the heels and toenails—areas that are often in contact with the ground surfaces and where cracks and grossly altered nail beds commonly appear. In later stages, changes in the skinfolds occur, followed by severe characteristic dermal alterations known as “mossy foot” and dermal nodules.^44^ Neurological changes can develop, including both sensory numbing, with loss of sensation that can lead to patients’ accidentally burning themselves in fires, and pain that is commonly localized to the foot of an affected leg. A discomforting pain described by patients as “deep scratching in their bones,” has also been reported in Tanzania (C. D. Mackenzie, unpublished data).

As already described, acute attacks (ADL) are important both in terms of patients’ resilience and in exacerbating the clinical condition. The duration of ADL attacks varies between 3 and 9 days,^45^^–^^48^ during which the patient is usually unable to perform regular work, thereby causing economic strain for the patients and their families. The ADL attacks involve various degrees of discomfort, including pain in the affected leg, feeling feverish, feeling cold, having limb and lymph node inflammation, and having swelling. In addition, depending on the severity of the infection, some patients may have a headache, vomiting, altered sensorium, and urine incontinence. Peeling of the skin (exfoliation) in the affected area can occur as the acute attack subsides, but exfoliation is generally not seen in less severe attacks. Once ADL has been treated and is subsiding, an increase from the pre-attack edema will persist, usually with additional dermal changes; without care, repeated attacks eventually lead to gross, chronic lymphedema. In all cases of an ADL attack, the presence of “entry lesions” (see below) should be sought and treated to promote their healing and recovery after the attack.

### Sequence of tissue-level changes.

**Figure 2.**
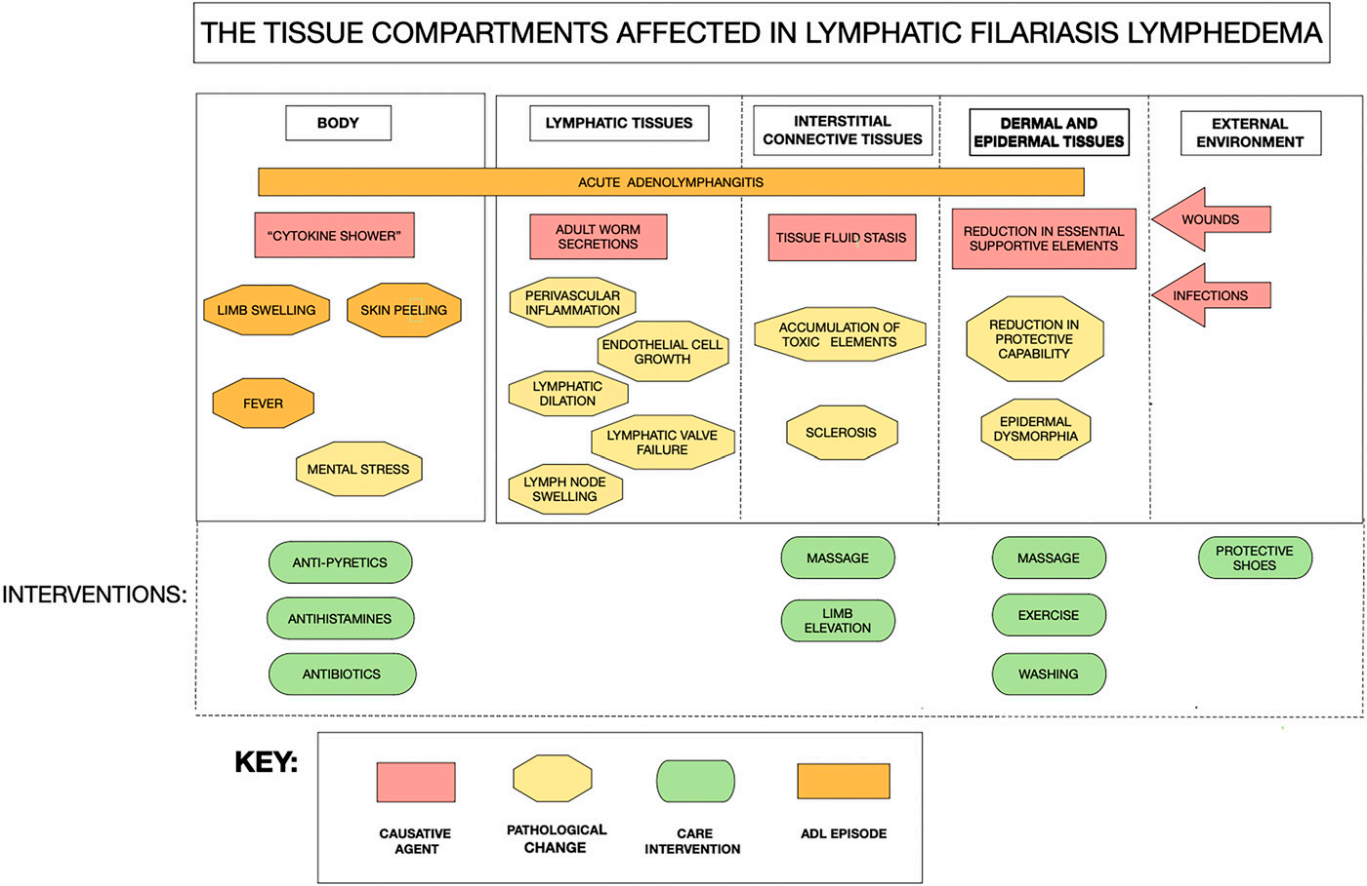
The tissue compartments involved in lymphatic filariasis lymphedema. The various causative and pathological elements contributing to the clinical changes and the target areas for care intervention. ADL = adenolymphangitis.

For optimizing treatment, it is important to understand the progressive changes of filarial lymphedema over time, from when the adult worm first takes up residence in the afferent lymphatic vessels of the lymph nodes, to the enlargement of the involved lymphatics, to the inflammatory changes associated with the presence of the adult worms and their secretions, and to the subsequent accumulation of lymphatic fluid in the associated interstitial connective tissues (Figure 2). These events are associated with progressive degeneration in the skin’s normal homeostatic and protective function, opening opportunities for the establishment of secondary bacterial and fungal infections with additional associated gross pathological changes. Indeed, it is these secondary infections, along with a compromised lymphatic system, that drive progression of lymphedema even in the absence of persistent filarial infection; this is particularly important because most lymphedema patients do become free of active filarial infection over time. Presumably, such individuals have built up a higher level of inflammatory immune responsiveness, which is effective at warding off new incoming filarial infectious larvae (the L3s), but at the cost of local inflammation that ultimately drives further vessel enlargement and worsening lymphatic function. Many studies have characterized the different immune profiles between lymphedema patients and those who have ongoing infection (microfilaremia) but without overt disease^49^^,^^50^; these observations suggest a significantly elevated pro-inflammatory immune response in patients with lymphedema compared with those without. Such a view of the pathogenesis of filarial lymphedema^51^ is useful both for providing appropriate care, independent of filarial infection status, and for understanding the pathological elements that might be targeted in investigations aimed at improving care, both before and after filariasis transmission has stopped in affected communities.

Clear differences exist in the histological changes induced by live or dead adult worms and by the earlier developmental stages, as documented in the lymphatics of experimental models and humans. Live worms induce inflammatory responses around the harboring vessels, likely stimulated by components in the worm’s secretions such as toll-like receptors ligands^52^^,^^53^ originating from the worm’s *Wolbachia* endosymbionts or other worm components.^54^ These responses include a cellular infiltration of monocytes, eosinophils, and lymphocytes, along with inflammatory mediators such as vascular endothelial growth factors (VEGF-A, VEGF-C and sVEGFR-3, VEGF-D), angiopoietins (Ang-1/Ang-2), fibroblast growth factor, and placental growth factor.^55^^,^^56^ These mediators, in concert, lead to excessive growth in the number of endothelial cells (lymphatic endothelial hyperplasia) and other cells in the vessel wall.^57^ It is this “overgrowth” that functionally disables its system of intra-lymphatic valves (essential for the directional flow of lymph fluid through the vessels) that is the major contributor to lymphatic functional insufficiency. Fluid stasis in the tissues eventually leads to pathological changes such as fibrosis, neuropathy, and compromised tissue remodeling. Because the affected subcutaneous tissues supply and support the adjacent skin layer (the dermis), their alteration and degeneration affect the integrity and biological functions (both homeostatic and immunological) of the skin itself. Thus, the progression of lymphedema evolves first from lymphatic alteration (a vascular condition) to major skin damage (a dermatological condition). Interestingly, the serum levels of several recognized stimulating growth factors also decreased in patients after successful treatment, even months before their lymphedema-stage reduction was observed clinically.^58^ Single-nucleotide polymorphisms in VEGF genes have also been associated with both lymphedema and hydrocele.^59^

### The role of secondary infections.

The lymphatic system is important in removing organisms (principally bacteria or fungi) that enter the body. Where there is lymphatic dysfunction, such organisms tend to remain in the static lymph fluid, grow in this supportive medium, and likely contribute to acute attacks. Entry lesions (wounds, lacerations, paronychia) and interdigital fungal infections are very common in patients with lymphedema, where the digits are often swollen, thereby reducing the interdigital space and encouraging moisture to persist, often facilitating fungal infections. Such infections are associated with pruritus, and the patient’s action of scratching with their fingernails or rubbing on rough surfaces can lead to entry lesions and their consequences. Moisture, as a factor that encourages fungal infections, is likely to contribute to the increase in co-infections reported during the rainy seasons in many areas where patients commonly wade through water; these patients frequently suffer recurrent episodes of acute attacks.^47^^,^^60^ Open wounds, which allow entry of secondary-infection organisms, are an important pathogenic factor in lymphedema, but there are few direct studies on the microbiological profiles of open wounds in LF lymphedema. Earlier assessment of anti-streptolysin O titers as a surrogate marker for streptococcal infection in patients with acute ADL found that these titers were persistently elevated in 90% of patients with wounds, a finding consistent at least with the notion that these wounds could have been portals of entry for *Streptococcus*.^60^

A seemingly fertile area of interest shaping the understanding and development of new treatment strategies in other medical fields is the difference in the microbiomes (biofilms) of an organ in health and disease. Differences in the dermal microbiome between normal and lymphedematous limbs have yet to be fully defined;^61^ however, when addressing questions of the microbial environment’s influence on lymphedema and, importantly, when selecting antibiotics and other antimicrobial agents for treatment, it is important to be cognizant of the environment in which the patient lives, works, and has other potential microbiological exposures. Recently, a study of the microbiome in the related lymphedema condition due to podoconiosis suggested a positive correlation between increasing lymphedema severity and noncommensal anaerobic bacteria (especially *Anaerococcus provencensis*) and a negative correlation with the presence of *Corynebacterium*, a constituent of normal skin flora.^62^ It remains to be seen which (if any) of the species identified plays a role in skin pathology or might even be found in patients’ blood during an acute attack.

## PROVIDING LYMPHEDEMA TREATMENT OF INDIVIDUALS AT THE CLINICAL LEVEL

There are two distinct contexts in which treatment of patients with LF lymphedema needs to be considered: 1) at a clinical level, where individual patients require specific treatment, and 2) at a public health level, where treatment is part of the strategy for reducing the disease in endemic populations where LF prevalence might be as low as 1–2%.

### Patient presentation and treatment strategies: Acute and chronic care.

**Figure 3.**
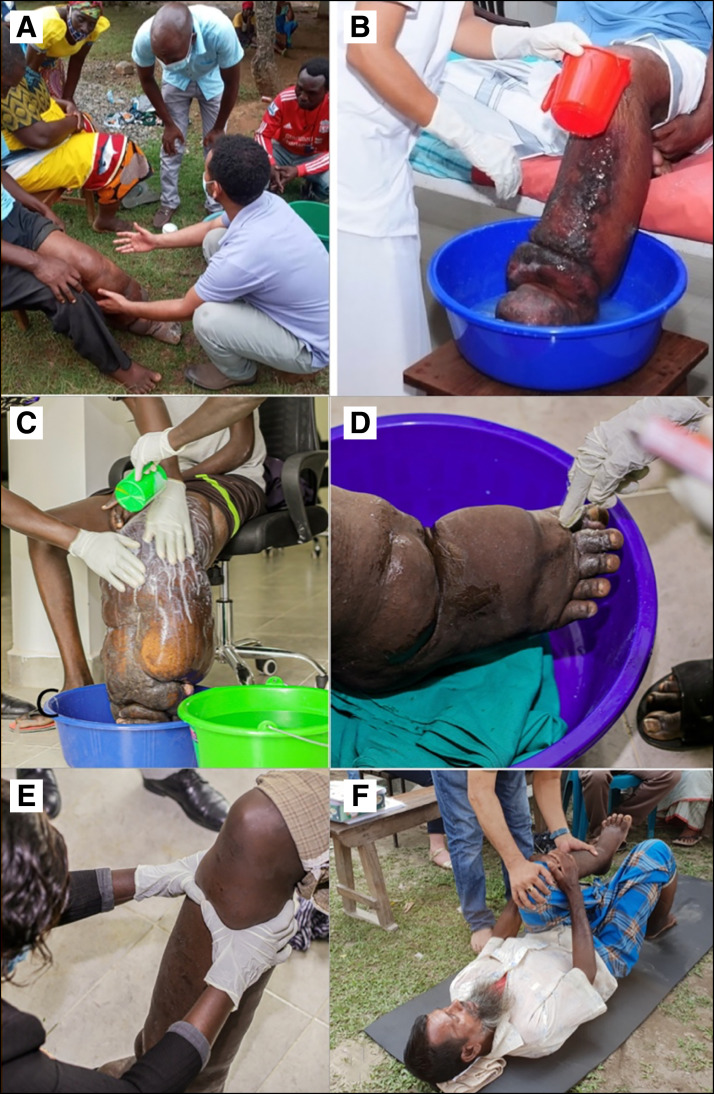
Examples of implementing elements of the Essential Package of Care (EPC). (**A**) Fikre Hailekiros (Ministry of Public Health) training healthcare workers on EPC in Malawi. (**B**) Applying water to a lymphedematous leg to begin the EPC in India (the hygiene component of EPC). (**C**) Washing the soap from an affected limb in South Sudan (the hygiene component of EPC). (**D**) Applying antibiotic cream to folds and interdigital areas of a patient in South Sudan (the wound care component). (**E**) Carrying out appropriate massage in South Sudan (addressing edema of the limb). (**F**) Sultan Mahmood (Ministry of Public Health) instructing physiotherapy procedures in Bangladesh (exercise and belly breathing component, etc.)

**Figure 4.**
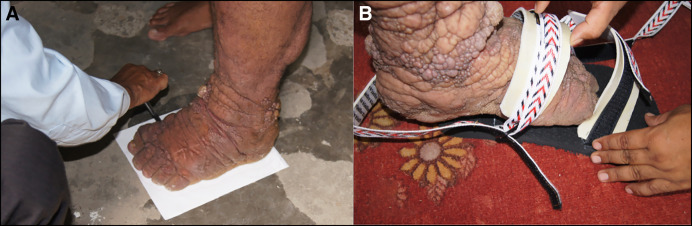
Constructing adaptable shoes for filariasis patients with lymphedema. (**A**) The shoemaker at the Lalgadh Leprosy Hospital, Madhesh Province, southern Nepal, draws an outline of the patient’s foot on the solid sole for the new shoe. (**B**) The completed adapted shoe can be seen with adjustable wide Velcro**^®^** bands that allow for changes in the size of the foot.

Many individuals first present to their health system for consultation about their lymphedema without concurrently having an ADL or an acute worsening of their symptoms. For them, immediate institution of a treatment strategy based on WHO’s recommended EPC for lymphedema management is most appropriate (Figure 1). The principal elements of the EPC include hygiene (washing of limbs every day), skin and wound care (including use of antifungal and antibiotic creams), exercise, elevation of the limbs, and wearing of comfortable and appropriate protective footwear (Figures 3 and 4).^63^^,^^64^ This approach has brought significant, albeit still poorly quantified, relief to patients with filarial lymphedema.^2^^,^^9^

Other affected individuals visit their clinics or hospitals because of an ongoing ADL attack or a sudden worsening of their lymphedema condition. For them, the immediate goal is to reduce the intensity of their ADL symptoms and to address their worsening lymphedema; the sequence of action for their care should be as follows:

- Immediate treatment with antibiotics, analgesics, antifungal creams, and guidance on other ADL alleviation methods (such as resting, drinking more water) and wound care. Undertaking such immediate care can be particularly challenging in many rural settings, as patients often need to travel to medical centers at the time when they are most reluctant or unable to move and walk long distances. When the antibiotic that targets the most probable infectious cause of the ADL is selected, it is useful to consider the local biogram and environment in which the patient lives and works, as well as any specific exposures or disease comorbidities they might have. Entry lesions should be identified and then be treated with antifungal, antiseptic, or antibiotic ointments; however, it is important that the MDA drugs (diethylcarbamazine, albendazole, ivermectin) not be administered during an acute attack, as the immediate goal of ADL treatment is to quell or minimize local inflammatory responses, not potentially trigger additional inflammation from any parasites that might be affected by the MDA drugs.
- Longer term treatment measures follow the EPC protocol on washing, exercise, elevation, physiotherapy, and even prophylactic antibiotics in some severe cases. Because it is a common belief that acute attacks follow some stress or injury to a lymphedematous limb, it is important that patients protect their affected limbs from trauma (e.g., by wearing shoes when walking or working in the field).

When deciding on the long-term treatment strategies for individual patients, it is important both to consider the time it has taken for the development of the pathological changes and to resist the tendency to regard all patients of roughly the same physical presentation as being clinically identical. Knowing the duration of the condition can significantly affect the emphasis placed on a particular component of the lymphedema management strategy (e.g., when there is a greater need for more physiotherapy or when making a prognosis for how much improvement can be expected).

Implementation of the EPC’s lymphedema management strategy should begin as soon as possible after the first signs and symptoms appear, especially in younger patients; healing and repair slow with aging in general, and this age-related slowing also likely applies to changes in older patients’ lymphedema. Thus, an individual’s age must always be considered when assessing the ongoing status or clinical progression of patients; an elderly person with long-standing lymphedematous and dermal changes is likely to respond less quickly to treatment than a younger individual with an equivalent lymphedema stage. Moreover, although the basis for treatment of most patients with lymphedema is the EPC, it should also be remembered that any comorbidities these patients have (e.g., diabetes, hypertension, obesity) also require specific medical attention in addition to that provided by the EPC protocol.

### Skin and wound care.

Carrying out daily hygiene procedures has been shown to be a most useful treatment to improve the health of the skin of patients with lymphedematous limbs, and this can also reduce the frequency and intensity of the most debilitating component of their lymphedematous condition, the acute attacks (ADL). Such procedures include washing the affected limbs with pH-neutral soap and water twice daily or at least once before going to bed. Even if only one limb is involved, both legs should be washed because there could already be lymphatic dysfunction in the apparently normal limb. Washing should not involve use of any hard objects such as a brush or scrubber because the resultant abrasion could precipitate an ADL attack. After the limbs are washed, the skin, especially between the toes and folds, should be dried with a clean cloth using a dabbing action rather than a potentially damaging swiping action. Clipping the nails at intervals, keeping the nails clean, preventing or promptly treating any local wounds or topical infections using antibiotic ointments, and applying antifungal ointment in the webs of the toes and in between deep folds to prevent interdigital and nail infections will help to prevent an ADL attack. In patients with late-stage lymphedema, proper local care of the limb may not always be possible owing to deep skinfolds or warty excrescences. In such patients, topical antifungal creams and even long-term oral antibiotic therapy with penicillin-type antibiotics is often recommended. Regular use of proper and comfortable footwear is an important element of lymphedema management and the prevention of lymphedema progression.

### Addressing aberrations in lymphatic drainage.

Fundamental damage to the lymphatics is an overdilation through vessel growth induced by inflammatory mediators, associated inflammation, and growth factors such as VEGF-A,^1^^,^^56^^,^^57^ with consequent loss of intraluminal valve function; it is not, as previously opined, caused by lymphatics that were “blocked with packed parasites.” Therefore, to prevent progression of the pathology, it is important that the lymph stasis be reduced as much as possible and that lymph flow be promoted, especially in the early stages of the developing condition. Three fundamental physiotherapeutic strategies are recommended.

#### Exercise and massage.

The affected area should be exercised regularly with movement of the joints to promote lymphatic flow and with limited exercise and movement of the limb, but not to a level that causes any untoward stress to the individual. This exercise can range from low-intensity movement of the affected limb and joints in situ to short walks in those with lower stage cases.^65^^–^^67^

Massaging the affected limb is useful, especially in lower stage cases where the tissues are more malleable.^68^ It is important that the massaging action be short in length, in a cranial direction beginning at the extremity, and using moderate pressure without causing pain. Massaging should be carried out twice a day where possible except during acute episodes.^69^ This procedure takes advantage of the natural anatomy of lymphatic vessels, their internal valvular system, and the movement of static lymph fluid in a cranial direction from one valvular unit to the next. It is effective because lymphatic vessels are not “blocked,” as the lymph stasis is due primarily to valvular insufficiency. Interestingly, the importance of massage is underscored by evidence that endothelial migration and neovascularization through new connective tissue can be enhanced by mechanical pressure and sheer stress.^70^

Additional efforts that are underway to enhance the impact of current lymphedema self-care measures include the addition of deep breathing, leg exercises performed in the supine position, and regular intake of drinking water. Significant improvement in lymphedema status and in reduction of acute attacks has been observed in both standard self-care (massaging that facilitates self-stimulated lymphatic drainage) and enhanced self-care groups of patients, but the biggest change observed was on legs affected by severe lymphedema in the enhanced self-care group.^68^ It still needs to be determined how successfully these and other such enhanced self-care measures can be integrated into standard self-care practices.

#### Resting position of the limb.

It is advantageous to keep the affected limb raised at night to enhance lymphatic drainage; this is best achieved by elevating the foot end of the bed using bricks or by placing a pillow under the mattress. Indeed, it is essential that the bed/mattress itself be elevated rather than having the patient simply place a pillow or cushion under the affected leg, as doing the latter may further reduce lymphatic flow in the affected limb during the night. Furthermore, a leg lying directly on top of a pillow or cushion could slip off during sleep and thus not be able to effectively enhance lymph flow. During the day, while the patient is sitting on the ground or in a chair, elevation of the limb is not optimal if it compromises regular movement and frequent exercising of the leg.

#### Deep breathing.

Regular sessions of deep breathing have been recommended^69^ and used in Bangladesh to good effect,^71^ and this form of self-treatment is thought to be advantageous in other forms of human lymphedematous conditions.^69^ Owing to the actions involved, this is also known as “deep belly breathing.”

### Adaptive shoe use.

It is essential to reduce injury to the affected limbs, and in the case of lymphedema of legs and feet, the most common form (i.e., providing protection to the feet through the use of adapted footwear) is vital (Figures 1 and 4). Strong soles of the shoe can be strapped to the foot using wide Velcro^®^ straps that accommodate the different sizes of the affected feet (Figure 4). This a situation where collaboration with leprosy control programs is useful, as many of these programs already have shoe production facilities.

### Anthelminthic treatment.

Ensuring removal of the offending agent that initially induced this lymphatic pathology (i.e., the filarial parasite) is essential, namely by introducing or continuing anthelminthic treatment, generally with the same very safe medicines used for the MDA phase of the LF program. Patients should also be provided with the anthelminthics used in the LF MDA program, albendazole and either ivermectin (where onchocerciasis exists) or diethylcarbamazine for non–onchocerciasis-endemic countries.^5^^,^^6^ It is important to note that anthelminthic treatment should not be given to a patient who is undergoing an active ADL attack, as this could exacerbate the attack and further seriously incapacitate the patient. Increasing the frequency of administration of these drugs to multiple times a year for individuals still exposed to infection has been advocated, though without carefully controlled comparative trials, especially for patients in the early stages of infection. In addition, doxycycline is now recognized as an effective macrofilaricidal treatment of LF because of its ability to deplete the essential *Wolbachia* endosymbionts in the adult-stage parasite; trials in Tanzania,^72^ Ghana,^73^ and India^74^ have all documented its efficacy when administered daily for 3–8 weeks. Interestingly, in studies in Ghana, doxycycline was also associated with grade-stage improvements in the lymphedema of patients when used with standard hygiene treatment, even in those who no longer appeared to harbor active infection.^75^ Such findings suggest that doxycycline might be exerting a direct effect on lymph vessel growth and proliferation. It was these and related findings that prompted the current trials previously described^76^ and the outcomes reported elsewhere in this Supplement to investigate whether and to what degree doxycycline might enhance the beneficial effect of the stringently applied hygiene measures of the WHO’s EPC for treating patients with filarial lymphedema.

### Surgical approaches to lymphedema reduction.

In certain countries where LF is endemic, surgical “debulking” is a treatment used for some cases of severe chronic lymphedema (“elephantiasis”),^77^^–^^79^ including the drastic step of amputation. However, in the long term, this surgical approach is most likely not beneficial, and it has been reported^80^ that after such procedures there has often been deterioration to an even greater degree of severe, chronic lymphedema than before surgery. One reason for this is that the dermal tissues in a lymphedematous limb are already recognized as being compromised in terms of their ability to maintain homeostasis; alterations ranging from delayed healing with persistent suppuration to overgrowth of collagen after surgery (keloid scars) indicate that the healing process in these patients is abnormal. Although debulking surgery is a common practice in certain countries, it has not been generally successful in endemic areas where healthcare is limited, and thus it is not universally recommended. Similarly, a more refined surgical procedure of “microsurgical lymphovenous shunting,” which creates an anastomosis between the lymphatic and blood vascular systems, was pioneered for nonfilarial lymphedema patients in Europe but has proven to be less satisfactory for treating the lymphedema caused by filarial infection; thus, it too has had limited long-term or widespread utilization.^81^^,^^82^

### Holistic approaches to lymphedema care.

Recognizing the importance of treating the whole individual rather than just the affected limbs is now very appropriately receiving appreciable emphasis. Aside from the obvious need to reduce the impact of stigma and comorbidities (e.g., diabetes mellitus, hypertension, and obesity), attention is being paid increasingly to improving patients’ mental health, especially related to depression. Integration of mental health services into neglected tropical disease (NTD) programs through strengthened collaboration at the community and primary healthcare levels is now actively recommended.^83^ Such attention has been especially catalyzed by collaboration through colleagues focused on leprosy care,^84^ and it has generally targeted issues of stigma reduction to address clinical depression and improve the QOL of these patients. The fact that advice is now available for healthcare workers in relation to NTDs such as LF^85^ and the realization that LF lymphedema care means more than simply reducing the obvious disease expressions of limb size or ADL frequency are reflected in current initiatives that address overall QOL for these patients and their families.^30^

The Ayurvedic medical approach, often in conjunction with yoga, has long been used by specialized practitioners in India and elsewhere as well as by dermatologists interested in treating chronic skin diseases including LF lymphedema.^86^^–^^88^ This approach, which includes attention to mental health, skin hygiene with topical emollients, limb compression, and physiotherapy, has been effective with good results in terms of reduction in inflammatory ADL episodes and in the size of affected limbs.^88^ However, as noted by those supporting this approach, further studies are needed to determine its practicality in healthcare-weak rural environments.

Recently, holistic integrated care packages of physical and psychosocial care for lower limb disorders caused by podoconiosis, LF, and leprosy were piloted in Ethiopia^89^ and Nepal.^90^ The care package involved a range of interventions at the levels of healthcare administration and both primary and community healthcare facilities. The integrated package resulted in significant improvements in dermatological QOL, self-reported disability, physical health outcomes (the number of acute attacks and leg/foot swellings), depression, stigma, discrimination, and community participation.^74^ It was found to be acceptable to patients, health professionals, and decision-makers. With the stakeholders being cautiously optimistic about such integrated lower limb disorder management, it has since been scaled up in Ethiopia.^91^ A feasibility study of integrating lymphedema self-care into self-help groups already supporting leprosy care suggested that although stigma could be a potential barrier, the attitude of the lymphedema patients toward availing such services can be very positive.^92^ Overall, integrated care for skin NTDs has progressed more slowly than that for integrated MDA, but the usefulness of such integration is so clear that it is worth addressing those elements principally responsible for holding this progression back. Those would be the lack of both baseline burden and cost-effectiveness data, the limitations of organizational structures and funding, and the complexities of integrated health-worker training.

### Effectiveness of treating individuals with LF lymphedema.

It is most important these days that both individuals with lymphedema and the health systems caring for them recognize that lymphedema is a condition that responds to good treatment. Indeed, although quantifying the effectiveness of implementing the EPC is an issue currently in need of improved long-term data, there is little doubt that implementing the EPC can be effective in preventing, or at least slowing, the worsening of lymphedematous changes, resulting in almost complete remission in low-stage cases.^69^^,^^93^ The EPC also has the important effect of reducing the number and severity of acute attacks and the severe consequences they can have.^94^ Although the continuing use of anthelminthic agents, at least in early infections, is also believed to be important,^5^^,^^38^ improvement associated with the EPC is most dramatic in those with shorter duration and lower stage conditions, though it can be seen even in patients with high-stage lymphedema.

### Considering approaches used in treating non-LF associated lymphedemas.

It is valuable for those providing lymphedema care in LF-endemic countries to be aware of the approaches to lymphedema care in non–LF-endemic countries, particularly so that they might expand the range of care they currently provide if new strategies look to be safe, practical, and of benefit to the patients.^95^^,^^96^

Because lymphedema is a disease caused by lymphatic functional insufficiency, the changes in affected tissues bear similarities across multiple causes. Use of treatments such as lymph node removal and radiation therapy for cancer is the greatest cause of lymphedema in affluent countries, and considerable research has been conducted on arm lymphedema among women who have undergone treatment of breast cancer.^97^^,^^98^ Although the incidence of breast malignancy continues to rise, the number of women experiencing arm lymphedema has significantly declined because of both an increase in the awareness of lymphedema among primary healthcare professionals (which has led to the use of lymph node–conserving surgery) and implementation of a prospective surveillance model for breast cancer patients, leading to the prevention of new lymphedema cases.

A prospective surveillance model aims to identify and then monitor patients at risk, applying preventive treatment if subclinical changes are detected. Monitoring is commonly carried out using self-reported symptoms confirmed by bio-impedance spectroscopy, which can detect small changes in the extracellular fluid load. If a low threshold of 3% is crossed, conservative therapy follows with the application of meticulous skin care, exercise, massage, and compression.^99^ Such a prospective surveillance model in LF-endemic regions could have a similar impact on reducing progression of at-risk cases to overt disease. Modifications of the present model would be needed to account for location-specific challenges in communities endemic for LF and podoconiosis, particularly the extension of existing case registration and monitoring tools to identify and include high-risk communities and at-risk members of the community. Because the increase in extracellular fluid load can often be felt as limb heaviness before apparent swelling, in the absence of objective measures anyone with a history of infection and subjective symptoms should be included in the education and support program. Early identification and intervention could also be implemented through schools in regions where transmission has not yet been broken or has only recently been stopped. Young people with latent disease have the best opportunity to reverse early changes and lead lives without lymphedema if they are educated in the necessary signs and intervention measures.

Just as with LF-induced lymphedema, any increase in awareness of the causes and management of lymphedema helps to reduce the mental health issues associated with stigma and social exclusion. Monitoring of cases can be performed by health workers trained in reliable and repeatable measures such as circumference and the “pitting” test for edema.^97^^,^^98^ These simple measures can determine the level of free fluid present for monitoring individualized self-care programs and offer realistic expectations on the potential to reverse limb size. The longer the disease has been present, the longer health service support will be required, but a prospective surveillance and early intervention strategies can minimize the number of new cases that require future care. As a rule, to reverse severe skin and tissue changes, daily treatment is required for at least 1 year for every year the swelling has been present, up to about 5 years, by which time everyone should see visible improvement.^4^ Nevertheless, stopping the pathological changes by adopting rigorous EPC is likely to have a positive effect on all individuals, with a more rapid positive effect in those with less advanced tissue changes than in those who have had the condition for longer terms and thus have more fibrosis and more dermal pathology. Thus, people who have had lymphedema for a long time need to be patient and maintain daily self-care even when they will not see much daily change. In contrast, those who have noticed swelling for only a few months or less may achieve complete resolution after a year or so of self-care and require no further treatment. Including people who have only mild swelling in lymphedema-care services and packages ensures that the future cost of lymphedema service delivery will be reduced.

Compression is still an essential component of best-practice lymphedema management, and a range of new devices and alternative systems are readily available for home use in affluent countries^100^; it was also suggested for lymphedema in filariasis as early as 1938.^101^ These new devices include self-adjustable wraps, which offer a user-friendly alternative to custom-made compression garments; they are durable, washable, and can be worn around the home even if outside use is still impractical.^102^ There are new-generation overnight garments, which are easy to doff and don and which provide gentle compression during sleeping. Although challenging, local provision of appropriate and acceptable compression therapy should be considered wherever possible, and given the right equipment, effective compression can be managed at home.^103^ It must nevertheless be recognized that it is often difficult to keep material garments clean in dusty and often wet rural environments,^4^ such as those where many LF-affected patients live, and this can contribute to inducing unwanted secondary infections and other hygiene difficulties. The difficulties of keeping compression bandages clean are especially challenging when they are used constantly and by individuals who are being encouraged to exercise, or who need to continue working in their fields, etc. Washable or disposable compression bandages need to be developed and tested for field use, and garment manufacturers could be encouraged to donate such products or materials and support the training of their use in national MMDP programs in the same way that drug companies have supported the MDA.^104^^,^^105^

Adhesive “kinesiology” tapes have been applied in breast cancer–related arm lymphedema as an alternative to a compression sleeve, with promising results.^106^ These elastic tapes are of a light cotton construction that has a thickness, weight, and elasticity similar to that of the skin, and by stretching and recoiling during movement they support and stimulate lymph formation and flow in superficial lymph vessels. The tape can remain on the skin for several days, and the adhesive is well tolerated by most people. Because they do not cover the skin completely and are made of a breathable material that dries quickly, kinesiology tapes may be an alternative to knitted compression garments in tropical climates; however, as a single-use medical item, the tape is expensive and produces nonrecyclable waste.

Surgical procedures either to improve lymphatic function or to remove excess tissue have also progressed in response to demand for better options among women with arm lymphedema.^77^^,^^107^ Lymph-venous anastomoses can be surgically created to redirect patent lymph vessels around an obstruction and are most successful in the early stages of lymphedema.^81^ Long-term follow-up suggests that many such anastomoses will fail over time as the fluid load reduces. Similarly, free lymph node transfer has been found to increase lymph drainage from the arm when included in a breast reconstruction, but this has not yet become standard practice and carries an increased risk that the mound will fail if the graft does not take properly. Specialized liposuction procedures have been developed to remove the fatty overgrowth typical in the middle stages in lymphedema arms and, more recently, legs.^108^ This form of liposuction requires comprehensive physiotherapy including compression bandaging prior to surgery to remove any free fluid and to soften fibrosis. Postsurgical physiotherapy and compression are also required.

## PROVIDING LYMPHEDEMA TREATMENT OF LF-ENDEMIC POPULATIONS AT A PROGRAMMATIC SCALE

### Challenges and strategies in lymphedema program management.

The basic principles for managing individual patients with lymphedema are clear, but to maximize “public health” progress in lymphedema management on a national scale, it is necessary to ensure that all those in need have both opportunity and access to the most appropriate treatments available. Such a public health dimension for patient management requires addressing a largely new set of variables with distinctly different “tools” to create new solutions. Indeed, although GPELF itself has achieved remarkable progress since its inception in 2000, this progress has been driven largely by the adoption and expansion of its MDA programs (with their distinct priorities, clear guidelines, and well-defined targets) aimed at interrupting LF transmission. However, for promoting the Program’s MMDP agenda and bringing it also to the fore, significant programmatic challenges still need to be identified and addressed directly.^2^

For example, before 2021 only 1.15 million cases of lymphedema, from an estimated total of 17 million cases, had been reported to the WHO by 61 of 72 LF-endemic countries, and only 10 of the countries had reported providing specific access to care for their affected patients.^21^ One important reason for the relatively slower (compared with MDA) progress in developing lymphedema interventions is a still incompletely developed programmatic structure for the MMDP effort, along with an absence of well-defined indicators and strategies for assessing morbidity itself, rather than just the availability of care, and tracking its alleviation. Tracking patients’ improvement through a yet to be officially defined set of criteria (e.g., reduction in ADL attacks, increase in mobility, and use of the WHO QOL indicators) would be very valuable for assessing programmatic progress as well as providing good advocacy for the LF program. Although the inclusion of such morbidity indicators has been repeatedly advocated for LF control/elimination programs for years, now that the current expansion of lymphedema care programs targets providing 100% geographic national coverage with access to appropriate lymphedema care for every needy patient, the creation of a specific MMDP framework with well-defined epidemiologic indicators has become an increasingly urgent priority.

To organize lymphedema management programs at a public health scale, three distinct but interrelated target “audiences” must be mobilized. At the core are the patients, whose numbers and needs for care must be defined and addressed; however, before addressing these patient needs, those responsible government organizations (i.e., the health systems already in place) and those organizations and institutions able to provide the necessary resources to initiate the MMDP programs (e.g., Non-Governmental Organizations, international agencies, donors) must be engaged as well. Indeed, to catalyze successful, sustainable lymphedema management programs, it is particularly important to ensure strong commitments from national governments and Ministries of Health. It is also imperative that stakeholders, spearheaded by the WHO, facilitate a policy framework within endemic countries that envisages 100% geographic coverage and universal accessibility to services in both current and formerly LF-endemic districts. Securing such commitment and involvement of key officials at both federal and provincial levels is essential for prioritizing the MMDP, for mobilizing adequate resources, for enhancing capacity and infrastructure, and for streamlining logistics. In the long term, it is essential that sustainable care for patients be primarily supported by national health systems, as envisioned in the WHO requirement for MMDP’s integration into public health systems. Currently however, most countries are transitioning from the initiation and early maintenance phase of LF care supported by NGOs, donors, and research organizations to the needed long-term involvement of national health systems. It is vital that this early MMDP support involves the training and empowerment of local health facilities and thus ensures the capability of long-term provision of LF care. Indeed, when these advocacy and long-term policy efforts have been carried out well, compelling examples of programmatic success have been seen in India,^109^ Bangladesh,^110^ Togo,^111^ and Ethiopia.^112^ Based on the experiences of such successful programs, along with extensive research carried out in many countries, the core elements of a comprehensive, public health lymphedema management strategy can be outlined in the following five essential activities (Table 1).

**Table 1:** On-the-ground elements of a programmatic lymphedema management strategy

Item No.	Area	Issue
1.1	Patient identification	Identify lymphedema patients by house-to-house morbidity census
1.2	Staff training	Provide at least one staff member in each facility with specialized training
1.3	Community awareness	Raise awareness in communities and patients of availability of lymphedema care at health facilities
1.4	Patient training	Provide counseling and training to patients on self-care techniques
1.5	Patient support	Establish support system to motivate patients and facilitate self-care

#### Patient identification.

As described above, identifying lymphedema patients through an HHMC is a key step in both giving all patients access to healthcare for their condition and monitoring the success of GPELF. By conducting HHMC, accurate information about lymphedema prevalence and distribution can be gathered that enables effective planning, estimation of medicine requirements, capacity building, and provision of informed services. Not only is the HHMC highly efficient and accurate,^113^^,^^114^ but approximately 65% of such identified patients are also in the early stages of lymphedema,^114^ thereby presenting an opportunity to potentially reverse or halt the progression of their disease. In contrast, burden assessments conducted through alternative methods (such as an add-on to MDAs) often result in detection of only a small proportion of cases^1^^,^^115^^,^^116^ and mostly miss those individuals with early-stage disease, thereby depriving them of the necessary care and support to prevent worsening of their condition. Although often assumed to be unfeasible and expensive, HHMC programs can also provide additional benefits including excellent training for health workers and volunteers, expanded implementation to cover progressively larger regions, integration with other healthcare activities (especially those related to other skin diseases) to improve cost-effectiveness, and opportunities to introduce and test innovative approaches (e.g., mobile phone–based technologies)^10^^,^^11^^,^^13^ to public health challenges. Indeed, such a targeted search for individuals affected by lymphedema carried out by community health volunteers reporting to a central base was shown to deliver 5–10 times more cases than when lymphedema cases were identified only as an adjunct to MDA.^10^

#### Staff training.

At least one staff member in each health facility should receive specialized training in lymphedema care, and volunteers should be enlisted from the community to support the program. A cascaded-training or training-of-trainers model can effectively disseminate lymphedema care instructions through multiple layers of the health system.^109^^,^^117^ Well-planned, standardized training instructions across all districts and health staff further enhance the value and impact of the training, as does having the required training manuals and information sheets prepared in local languages.

Particularly important for effective training is staff participation with patients during live demonstrations of lymphedema management practices, such as washing, exercises, and massage, to provide hands-on experience to the trainees. The incorporation of village volunteers who are already engaged in other health programs would be optimal for scaling up and sustaining the program. Considering the constraints of cost and time, the training provided to village volunteers may not need to be as extensive as for other health workers. However, it should cover the basic mechanisms of the disease and simple management concepts, and most importantly, it should emphasize that self-care and lymphedema management are affordable, effective, and easy to administer.

Ensuring that knowledge about how to provide care for lymphedema remains in the community health system and with the patients themselves can be a major challenge. Health center staff change, patients become distracted from self-care, and the number of cases and their severity reduce over time as transmission wanes. To counter the impact of these issues on maintaining active MMDP activities, it is important to monitor the compliance of patients in carrying out the EPC and to provide refresher training sessions to health workers and patients where needed. The availability of training material (protocol sheets, instruction videos, etc.) in local health centers can help maintain awareness of the MMDP care procedures. Nevertheless, determining the optimal ways to maintain EPC programs in different country settings is an area that needs more active investigation.

#### Community awareness.

Raising awareness among communities and patients about the availability of care at health facilities is critical. When health workers and volunteers inform affected families and spread the word in communities about the simple and inexpensive lymphedema management practices available, they help to create optimism and a positive attitude toward visiting health facilities and seeking training in lymphedema management.^9^ Successful publicity campaigns to reduce stigma and to raise awareness and utilization of treatment centers can be achieved by displaying posters and banners at community centers, hospitals, and other healthcare facilities.

#### Patient training.

Counseling and training on self-care techniques should be provided to patients. To ensure comprehensive coverage and to reach every patient requires two complementary approaches by health workers: 1) providing services at health centers and 2) offering “doorstep assistance” to patients. During patient contact and interaction, health workers should take the opportunity to provide counseling, explain the underlying reasons for the patient’s condition, and emphasize the potential consequences of untreated or poorly managed lymphedema. By receiving this essential information, patients gain a better understanding of the importance of adhering to lymphedema management practices and the potential negative outcomes of neglecting their condition. Demonstrating these practices, such as leg washing, elevation, and exercises, is important to ensure proper understanding and implementation. It is equally essential to explain the importance of protecting the affected area from injuries and discouraging harmful practices. During such demonstrations, it is useful to include a family member or friend so they will be able to help the patient during the washing; their engagement will also help reduce stigma in the long run. By highlighting the simplicity and cost-effectiveness of these techniques, patients are much more likely to feel motivated and become empowered to participate in their own care.

Every effort should be made to facilitate patient self-care, including provision of repeated training sessions, necessary supplies (e.g., soap, towel, bowl, gauze cloth), and clear guidance (ideally in the form of laminated pamphlets depicting the steps for good lymphedema management), with practical tips to enhance patients’ understanding and empower them to take ownership of their management. To optimize time and resources, patients can be trained in groups, which allows not only for simultaneous education of multiple individuals but also for fostering peer support and creating a sense of community and reinforcement among patients with similar experiences.

#### Patient support.

A support system to motivate patients and facilitate the transition to family-based self-care should be established. For health workers responsible for creating a comprehensive lymphedema management strategy, building a robust patient support system is essential. Such a system includes family members and community volunteers who can provide lymphedema patients (particularly older patients and those in advanced stages of disease) with the assistance they need while also strengthening their motivation and psychological well-being. Such support groups and networks enable patients to effectively implement and sustain the recommended hygiene and self-care measures^2^^,^^102^ and, ultimately, empower them with only limited help from family members to take charge of their own care and thereby enhance their own independence and long-term well-being.

### Maintaining quality MMDP activities.

One of the most challenging problems that countries face, despite their best intentions, is maintaining long-term quality in their lymphedema care initiatives (Table 2).

**Table 2:** Challenges to be addressed in maintaining quality MMDP care activities

Item No.	Issue
2.1	Sustainability in ensuring that patients maintain use of the self-care package over time
2.2	Quality Control: ensure that the self-care that is instructed, provided, and supported is of High Quality, and that it is practical for the particular environment in which the patients reside
2.3	Utilizing Partnerships to enhance the success of national MMDP programs at all levels of the medical, research, and civil communities
2.4	Integration with in-country groups carrying out similar activities that can be challenging but that are often of mutual advantage to each group

MMDP = morbidity management and disability prevention.

#### Sustainability.

Sustainability of lymphedema management programs, at both individual and community levels, provides major challenges that can be affected by many different concerns, including the perceived and actual costs and benefits of self-care accrued to the patients, the availability of support from volunteers and family members, and the affordability of necessary supplies. At the individual level, behavioral change introduced by good counseling and training to practice self-care, frequent encouragement of patients by volunteers and family members, and high-quality training of patients all have strong potential to improve adherence to, and sustainability of, self-care. At the community, district, and province levels, good leadership, program monitoring and evaluation, appropriate corrective steps, good supervision, regular training of health workers, uninterrupted provision of commodities to health centers, and updating of registers are all demonstrably important in sustaining MMDP programs.^117^ The costs of such programs are generally low, mostly for training, health education, and materials for washing. They can decrease significantly after the first year of an MMDP program^118^^,^^119^; one study in India even identified “per-person savings” as being 185 times the program’s “per-person cost.”^120^

Still, sustaining the protocols over an extended period has been challenging for many lymphedema programs for a variety of reasons; these relate to the lack of needed medicines, the cost of soaps, patients’ long-term dependence on caregivers or assistants, and the competing needs occasioned by the rural and often impoverished environments in which the patients live and work. Even keeping up with the hygiene protocol is often a challenge for individuals who work in rural environments such as vegetable, fruit, and rice farms, where they are unable to maintain a strict regimen of hygiene and often subject their limbs to trauma and an unclean environment. Programs in some countries have supported patients in forming “self-help” groups for helping each other initiate and continue to use the best hygiene practices, as well as for sharing medicines where appropriate; such “Hope Clubs”^121^ also provide an important opportunity to reduce stigma. Optimal ways of helping patients sustain their adherence to the EPC need to be investigated, as well as the reasons why patients do not always adhere to this protocol.

#### Quality control.

Before countries can be formally acknowledged to have eliminated LF, the WHO’s GPELF requires that they critically evaluate their lymphedema care services to ensure their quality and to share specific details about lymphedema care activities. A management tool (the direct inspection protocol) provides the framework for use in assessing the readiness of health facilities to provide quality lymphedema care through the collection of information on 14 individual factors. The tool has already been used successfully in Vietnam^122^ and Bangladesh^123^ to identify and remedy shortfalls in the availability of care in their MMDP programs, and it is currently being refined further for introduction into other national programs as well.

#### Partnerships.

Partnerships have played an essential role in initiating, expanding, and intensifying the lymphedema management programs of many countries. They have operated at essentially all program levels, including operational research, technical support, resource mobilization, scaling-up, and innovation. Both local and global partners have worked together to design, fund, and scale-up lymphedema management programs to meet the WHO requirements for countries to be recognized as having eliminated LF as a public health problem.^124^ It is essential that such partnerships continue to support these critical MMDP activities.

#### Integrated approaches.

Programs that focus on individual patient care can be “integrated” effectively with other programs by taking advantage of similarities of target populations, types and timing of intervention, means of delivery, training of health workers, case detection, and many other common aspects of program management. The principal purpose of such integration is usually to increase programmatic efficiency and cost-effectiveness. Successful examples of integration with LF lymphedema programs have been documented in southern Nepal (with a leprosy program^90^) and in Ethiopia (with a podoconiosis program).^91^^,^^125^

### Monitoring impact and progress of MMDP programs.

Successful MMDP programs have been established in many countries (e.g., Brazil, Bangladesh, Thailand, and Vietnam), and these are documented through their formal achievement of the WHO’s criteria for verification of “elimination of LF as a public health problem.”^8^ However, the tools currently available for monitoring the progress of MMDP programs remain relatively crude (i.e., recording the number of lymphedema patients identified, the number of local healthcare facilities enlisted and trained for service delivery, the quality of the services assessed, and for hydroceles, the number of surgeries performed). Unfortunately, there is no simple indicator of the impact that these activities might be having. Although research has shown that hygiene protocols do reduce the likelihood of lymphedema progression in most patients and in many cases improve the lymphedema and reduce both the frequency and intensity of acute attacks,^4^^,^^63^^,^^93^ charting the changes in individuals over time would, if available, be critical new information of value not only to the patient but also for demonstrating both programmatic impact and, importantly, the reduction or complete absence of new cases after successful implementation of the MDA. The ideal approaches and indicators to follow have yet to be defined, but determining the optimal indicators for use in an endemic setting is an important research goal for the program. As described previously (ref. 9), these might include the number of new cases that have developed after successful reduction in transmission in an area and measures of patient mobility and capability to carry out physical activities (often those related to earning an income), along with the more commonly measured changes in lymphedema stage and ADL frequency and severity. An increase in the ability of wounds in the skin of the affected limb to heal may also be an important indicator of improvement based on the observations of a reduction in dermal healing that has occurred in untreated cases (C. D. Mackenzie, unpublished data). Further investigation into this issue is needed to determine the usefulness of this parameter as an indicator of improvement.

## AREAS WHERE RESEARCH IS NEEDED

Improving the overall strategy for care of those with lymphedema is particularly challenging when targeting patients with filarial lymphedema who often live in areas with minimal access to medical care. The need for new investigations falls principally into two categories: those targeting better individual treatment and evaluation for affected individuals and those focused on improving program management for the provision of care to affected populations.

### Research aimed at improving lymphedema treatment in affected individuals.

Important questions related to individual patients are highlighted in Table 3, including the following:

**Table 3:** Priority needs for research aimed at improving treatment of lymphedema in affected individuals

Item No.	Issue
3.1	Understanding of Lymphedema Pathogenesis (including parasite, host, and tissue changes)
3.2	Identification of Targets and Tools to improve clinical staging and management of lymphedema (from LF and non-LF causes)
3.3	Role of Secondary Infections (bacterial, fungal) in the pathogenesis of LF lymphedema and best strategies to diagnose, treat, and prevent them
3.4	Dermal Dysfunction and degeneration: clarifying mechanisms and strategies for optimal use of topical and compression treatments
3.5	Development of more robust, precise Tools to Assess/Monitor Changes (including clinical, health burden, and QOL) in patients with lymphedema

LF = lymphatic filariasis; QOL = quality of life.

#### Pathogenesis.

Because strategies for improving lymphedema therapy are likely to emerge from a better understanding of the disease itself, study of its pathogenesis and immunopathology in animal models and human patients is essential; it is, however, also important to study patients with lymphedema of nonparasite etiology to identify potential new targets or mechanisms that could improve treatment of lymphedema from any cause, especially in the early stages of the condition. Indeed, it is incumbent on those focused on lymphedema due to LF to also be aware of research and improvements being developed for nonfilarial lymphedema as well.

#### Improved clinical tools.

Importantly, increased understanding of both the pathogenesis of lymphedema and its clinical response to treatments requires development of better tools to assess and monitor changes in individual patients. For example, assessing a lymphedematous limb using new noninvasive imaging technologies (e.g., thermography and magnetic resonance imaging^92^^,^^126^) can help to relate observed changes to ongoing clinical signs and symptoms; indeed, repeated observations over time are likely to be particularly informative compared with assessments made just at single time points. Investigating the dermal changes using newer noninvasive techniques such as optical coherence tomography are likely to be informative,^127^ especially as standard dermal biopsies are ill-advised owing to the altered healing processes present in lymphedematous skin.

#### Secondary infections.

Because secondary infections of lymphedematous tissue with bacteria and other organisms are important in the pathogenesis of lymphedema, a clearer picture of the microbial agents involved could lead to improved chemotherapeutic approaches to treating lymphedema, whether initiated by LF or not. Although numerous studies have shown the involvement of bacteria in the pathogenesis and development of LF lymphedema, there remain many unanswered questions about the role of bacteria at potential entry sites in triggering acute filarial attacks and even septicemia, as well as about the role of fungi in deteriorating skin cracks and wounds. There also remain important questions about optimal antibiotic and antifungal treatments that should be recommended in such cases.

#### Dermal dysfunction.

Despite recent increased interest in the role that the skin’s microbiome (biofilm) plays in maintaining skin health,^128^^,^^129^ investigation of potential skin biofilm differences between lymphedematous and normal limbs remains minimal, though the answers could well lead to better understanding and treatment of the dermal changes in the future. Furthermore, because alterations in the skin are a major consequence of lymphedema changes, the extent of the loss of the skin’s homeostatic capability, the degree of dermal dysfunction or degenerated function, and how these changes can be reversed are all prime areas for research. The optimal topical treatments need to be defined. In many countries, patients have reported good results from using locally available oils (such as coconut or palm oil) after their regular limb washing. Because the skin of lymphedematous limbs is compromised in its ability to function fully, it is likely that topical treatments will need to cover the entire affected area, not just the obvious area of wounds, skin cracks, and toenails. Simple, safe treatments such as these locally available agents are important targets for well-structured treatment trials. Agents that are cheap and easily applied, such as hypochlorous acid,^130^ and that have a positive effect on both improving healing and skin homeostasis have shown promise in pilot studies but need further investigation.

The use of compression as a therapeutic procedure is an issue that needs reevaluation through research. This is not often a recommended procedure for lymphedema patients residing in locations where it might be difficult to maintain a supply of clean compression materials, as there may be a danger of increasing contamination of the affected skin. However, as the use of compression is well known to be of value in reducing the accumulation of fluid in the affected limbs, it would be useful to investigate the development of ways to maintain the cleanliness and reusability of the materials used for compression.

### Assessment of clinical changes.

Just as advances in understanding the dermal and infectious determinants of pathogenesis would greatly benefit from better tools to define and measure changes in patients’ physical consequences of lymphedema, so also more effective and appropriate tools are essential for evaluating and tracking the QOL and health burden of affected patients. Such tools, tailored to reflect patients’ special clinical and social circumstances, are likely to provide highly relevant data about the lives of those with lymphedema and the effects resulting from any new treatment approaches. Recent comparisons of different QOL surveys focusing on aspects specific to LF have shown how important it is to increase our understanding of the effects that lymphedema can have on an individual.^33^ A better understanding of these effects, probably gained through a series of assessments in different study sites, will better define the questions that should be included in an optimal QOL tool; certainly, important regional and country differences will be identified.

### Research targets to improve lymphedema care at the programmatic level.

The most important programmatic research challenges are those that focus on keeping GPELF (with its MMDP targets for lymphedema and hydrocele management) afloat and functional, both operationally and financially. These are outlined in Table 4.

**Table 4:** Priority needs for research toward improving programmatic approaches to lymphedema care

Item No.	Issue
4.1	Identification of the Most Successful Strategies to Integrate LF Lymphedema Management with Other Health Programs
4.2	Determination of the Clinical and Economic Costs and Impact of the GPELF and its MMDP Initiatives
4.3	Refinement of the Instructions and Tools to Address Both MMDP Progress and Component Elements of the Programmatic Dossiers Required for Completion by National Programs to be Recognized by the WHO as Having Eliminated LF as a Public Health Problem
4.4	Mechanisms to Ensure that All Patients with Lymphedema Have Access to Adequate Care from the National Health System
4.5	Identification of the Most Effective Strategies to Facilitate Long-Term Sustainability of Patients’ Self-Care Regimens for Managing Lymphedema

GPELF = Global Program to Eliminate Lymphatic Filariasis; LF = lymphatic filariasis; MMDP = morbidity management and disability prevention.

#### Integration.

The GPELF activities that are focused on MDA are now also frequently key elements of other well-established public health initiatives. Indeed, active efforts to integrate these GPELF MDA activities with those of similar initiatives targeting either related diseases or diseases affecting related populations have proven to be cost-effective, successful, and increasingly popular. The GPELF initiatives focused on MMDP, however, have been more difficult to establish and more challenging to integrate. Research into different strategies for integrating these MMDP components with those of potentially related public health programs could greatly enhance the prospects both for broader understanding and for wider distribution of lymphedema management principles for affected populations. Indeed, it is important to understand how care for LF patients can best be maintained in the long term and how this care could, or perhaps should not, be integrated into the universal health coverage approaches within an endemic country’s healthcare system. Should separate insurance policies for long-term LF patients be developed? Where can the care for LF patients be most appropriately integrated with that of other conditions (leprosy, mycetoma, podoconiosis, and the like)?

#### Cost benefit.

Although lymphedema management costs themselves are low, support for their funding still depends on recognition of their value by national governments and external funders who must choose among competing priorities. For the GPELF, there is already clear evidence of very favorable benefit-to-cost ratios, but additional research is still needed to define both the clinical (physical, psychological, and QOL) and economic impacts of effective lymphedema management programs. Such information could greatly strengthen the advocacy messages that are so important for donors and government agencies to justify their investments in MMDP.

#### Monitoring MMDP efforts.

Just as the clinical indicators of change in LF lymphedema over time (with and without treatment) need further definition and testing, so too do the programmatic indicators. The suggested tools for documenting specific outcomes of programmatic MMDP efforts are already identified in available WHO guidance^131^ for preparing “dossiers” that summarize what national programs must achieve to be recognized by the WHO as having eliminated LF as a public health problem. Research that compares and defines more precisely the indicators that are most programmatically useful among those already proposed could identify practical tools and targets that would both streamline and promote programmatic attention to the lymphedema management needs of patients in affected populations.

#### Patient compliance/self-care sustainability.

Although essential for both programmatic and patient care success, sustaining lymphedema management programs over an extended period has been challenging for a variety of reasons; these include lack of medicines, cost of soaps, long-term dependence on caregivers, and many other competing needs. However, because patient self-care is at the core of the WHO’s strategy (EPC) for lymphedema management, it is essential that the programs be designed to facilitate patient compliance with the available guidelines in as many ways as possible. Research studies of the programmatic elements affecting patient participation at every step of the way, either facilitating or inhibiting it and relating either to public issues or personal concerns, should appreciably help to identify and overcome unnecessary barriers to patients engaging with the long-term treatment programs that they need in order to manage their lymphedema successfully.

## COMMENTS

The GPELF exists because there are people afflicted by LF disease. Success for this global effort has been defined as a world

- *without* new (or at least with very few) LF lymphedema cases because the transmission of LF infections has been reduced to very low (or zero) levels after successful anthelminthic treatment of all endemic areas
  and
- *with* the assurance that optimal clinical care will be available for all those already affected.

Although there can be little doubt that achieving GPELF’s goals is challenging, there remains an ethical obligation for the NTD community to continue to take every step possible to provide necessary care and improve the QOL of those affected by the clinical consequences of LF.

As the MDA component of GPELF winds down successfully in many countries, it is essential to hold discussions within each country to identify the best way to manage and implement their national lymphedema (and hydrocele) management activities to meet the specific WHO dossier targets for success. In such discussions, high-level Ministry of Health personnel and those of other political bodies must be involved. It is most important that public health systems and their nongovernmental partners provide care for those with residual disease from earlier infections who will need care long after the MDA has been successful and has stopped. Innovative ways of providing such long-term care for those severely affected need to be developed, perhaps through separate, specifically targeted national or international funding efforts.

The current GPELF requirements for documenting a successful national LF elimination program (in an official dossier^131^) were developed with a focus on both what was a practical possibility and what could provide some still-to-be-defined adequate level of care to patients living in locations without easy access to healthcare. There has always been greater focus on the MDA component of GPELF than on MMDP, and even within MMDP it has been easier to focus on providing hydrocele surgery than to care for lymphedema. It is very appropriate now that national LF programs and the supporting research community increase their attention on those individuals with lymphedema and find ways to strengthen the relevant components of the WHO’s current EPC and its implementation.

## Footnotes

^†^
It should be noted that the term “elephantiasis” is itself recognized as a stigmatizing term to those affected; there is currently a concerted effort to avoid using the term wherever possible.
